# Supplementary material for: A Randomized, Controlled Trial of Vitamin D Supplementation on Cardiovascular Risk Factors, Hormones, and Liver Markers in Women with Polycystic Ovary Syndrome
Source: Nutrients. 2019 Jan 17;11(1):188. doi: 10.3390/nu11010188 (PMC6356309; doi:10.3390/nu11010188)
Supplement: Supplementary file 1 [file nutrients-11-00188-s001.pdf]

Table S1. Between group comparisons of cardiovascular risk factors, hormones, and liver markers after vitamin D or placebo supplementation after excluding participants on metformin in the vitamin D group (n = 3) and the placebo group (n = 8).

	% Change		
**Parameter**	**Vitamin D (n = 15)**	**Placebo (n = 11)**	***p* value**
Weight (kg)	0.4 ± 3.9	−0.6 ± 2.5	0.12
BMI (kg/m^2)	0.4 ± 3.9	−0.6 ± 2.5	0.12
SBP (mmHg)	0.5 ± 6.5	3.0 ± 6.9	0.35
DBP (mmHg)	4.1 ± 10.4	1.4 ± 6.7	0.55
hs-CRP (mg/L)	7.5 ± 60.4	6.8 ± 56.6	0.98
TC (mmol/L)	5.0 ± 11.1	−1.0 ± 11.6	0.20
LDL-C (mmol/L)	6.0 ± 17.8	−0.4 ± 18.1	0.38
HDL-C (mmol/L)	0.7 ± 11.4	−3.2 ± 6.4	0.72
TG (mmol/L)	10.4 ± 23.8	15.4 ± 35.9	1.0
Fasting glucose (mmol/L)	3.1 ± 9.3	0.6 ± 9.0	0.51
Fasting insulin (μIU/mL)	15.6 ± 41.8	20.0 ± 48.4	0.81
HOMA-IR	−21.7 ± 51.5	23.4 ± 60.1	0.055
FAI	9.6 ± 73.1	−3.4± 26.5	0.58
Testosterone (nmol/L)	2.0± 48.9	7.3 ± 29.0	0.75
SHBG (nmol/L)	0.1 ± 17.2	11.6 ± 12.7	0.073
ALT (IU/L)	−15.0 ± 28.1	19.9 ± 24.8	**0.005** *
HA (ng/mL)	−19.2 ± 50.6	−16.0 ± 38.9	0.86
PIIINP (ng/mL)	−11.1 ± 33.4	0.2 ± 33.0	0.41
TIMP-1 (ng/mL)	−11.2 ± 34.2	−3.4 ± 36.6	0.91
ELF Score	−6.4 ± 10.9	−3.5 ± 10.9	0.49

Data are presented as mean ± SD. Bold data indicate statistical significant p-values; *$p < 0.05$, significant difference between groups. 25OHD: 25-hydroxyvitamin D; BMI: body mass index; SBP: systolic blood pressure; DBP: diastolic blood pressure; *hs*-CRP: high sensitivity-C-reactive protein; TC: total cholesterol; LDL-C: low density lipoprotein cholesterol; HDL: high density lipoprotein cholesterol; TG: triglycerides; FAI: free androgen index; SHBG: sex hormone binding globulin; HOMA-IR: homeostatic model assessment of insulin resistance; ALT: alanine aminotransferase HA: hyaluronic acid; PIIINP: amino-terminal propeptide of type III procollagen; TIMP-1: tissue inhibitor of metallo-proteinases-1; ELF Score: enhanced liver fibrosis.
